# Quantitative nuclear histomorphometry predicts oncotype DX risk categories for early stage ER+ breast cancer

**DOI:** 10.1186/s12885-018-4448-9

**Published:** 2018-05-30

**Authors:** Jon Whitney, German Corredor, Andrew Janowczyk, Shridar Ganesan, Scott Doyle, John Tomaszewski, Michael Feldman, Hannah Gilmore, Anant Madabhushi

**Affiliations:** 1 Department of Biomedical Engineering, Case Western Reserve University, 2071 Martin Luther King Drive, Cleveland, OH 44106-7207 USA; 2 Universidad Nacional de Colombia, Bogotá D.C, Colombia; 3 Department of Medicine, Division of Medical Oncology, Rutgers Robert Wood Johnson Medical School, Rutgers Cancer Institute of New Jersey, 195 Little Albany Street, New Brunswick, NJ 08903 USA; 4 SUNY at the University at Buffalo, 3435 Main Street, Buffalo, NY USA; 5 Department of Pathology, University of Pennsylvania Perelman School of Medicine, Philadelphia, PA USA; 6 Department of Pathology, University Hospitals, Cleveland Medical Center and Case Western Reserve University, Cleveland, OH 44106 USA

## Abstract

**Background:**

Gene-expression companion diagnostic tests, such as the Oncotype DX test, assess the risk of early stage Estrogen receptor (ER) positive (+) breast cancers, and guide clinicians in the decision of whether or not to use chemotherapy. However, these tests are typically expensive, time consuming, and tissue-destructive.

**Methods:**

In this paper, we evaluate the ability of computer-extracted nuclear morphology features from routine hematoxylin and eosin (H&E) stained images of 178 early stage ER+ breast cancer patients to predict corresponding risk categories derived using the Oncotype DX test. A total of 216 features corresponding to the nuclear shape and architecture categories from each of the pathologic images were extracted and four feature selection schemes: Ranksum, Principal Component Analysis with Variable Importance on Projection (PCA-VIP), Maximum-Relevance, Minimum Redundancy Mutual Information Difference (MRMR MID), and Maximum-Relevance, Minimum Redundancy - Mutual Information Quotient (MRMR MIQ), were employed to identify the most discriminating features. These features were employed to train 4 machine learning classifiers: Random Forest, Neural Network, Support Vector Machine, and Linear Discriminant Analysis, via 3-fold cross validation.

**Results:**

The four sets of risk categories, and the top Area Under the receiver operating characteristic Curve (AUC) machine classifier performances were: 1) Low ODx and Low mBR grade vs. High ODx and High mBR grade (Low-Low vs. High-High) (AUC = 0.83), 2) Low ODx vs. High ODx (AUC = 0.72), 3) Low ODx vs. Intermediate and High ODx (AUC = 0.58), and 4) Low and Intermediate ODx vs. High ODx (AUC = 0.65). Trained models were tested independent validation set of 53 cases which comprised of Low and High ODx risk, and demonstrated per-patient accuracies ranging from 75 to 86%.

**Conclusion:**

Our results suggest that computerized image analysis of digitized H&E pathology images of early stage ER+ breast cancer might be able predict the corresponding Oncotype DX risk categories.

**Electronic supplementary material:**

The online version of this article (10.1186/s12885-018-4448-9) contains supplementary material, which is available to authorized users.

## Background

Estrogen Receptor positive (ER+) breast cancers are a common subtype of breast cancer that can frequently be effectively treated using hormonal therapy if deemed to have a low risk of recurrence. However, early stage ER+ breast cancers that are at high risk of recurrence are typically treated with adjuvant chemotherapy in addition to hormonal therapy. While chemotherapy increases survival rates by reducing rates of recurrence in these high risk subgroups [1], there may be significant side effects including loss of hair, taste, cognitive function, and additional extensive medical care [2]. As such, it is critical to be able to determine the level of recurrence risk to plan treatment effectively so that the toxic side effects of chemotherapy can be avoided in low-risk patients.

Several methods of assessing tumor risk have been developed, including gene assays such as the Oncotype DX (ODx) Recurrence score, that stratify patients based on their risk of cancer recurrence [3]. The ODx test is a 21 gene assay that is currently employed for separating breast cancer patients into low and high risk of recurrence categories to help a clinician decide whether or not to prescribe adjuvant chemotherapy for early stage ER+ breast cancers [4]. The recurrence score is derived from the expression levels of multiple cancer-related genes, and ranges from 0 to 100 [4]. Patients with an ODx score of 17 or below are in the low-risk category, patients with ODx scores between 18 and 30 were considered intermediate risk, and scores 31 and above are in the high ODx risk category [5]. Unfortunately, Oncotype DX and similar companion diagnostic tests (e.g. Mammaprint [6], PAM50 [7]) tend to be expensive and time consuming due to the need for physical shipping of tissue samples to proprietary testing facilities. They are also tissue-destructive, making additional evaluation of other biomarkers or genes difficult.

The modified Bloom Richardson (mBR) grading scale is based on measuring nuclear grade (variation in nuclear shape and size), mitotic count, and tubule density. Each of these individual histologic primitives are assigned a score from 1 to 3 and then added to generate the cumulative mBR grade. Mina et al. [8] showed that mBR grade was also highly correlated the expression of proliferation genes used in the determination of ODx risk categories, and Flanagan et al. [9] identified a positive correlation between ODx risk category and nuclear grade when creating a predictive model of ODx based off clinical variables. Unfortunately, pathologic assessments of tumor grade are known to suffer from inter-observer variability [10].

Quantitative histomorphometry (QH) refers to the use of computer-aided image analysis of digitized pathology images to “unlock” more revealing sub-visual attributes about tumor morphology, which can possibly be correlated with disease recurrence independent of other clinical and pathologic features. These features might also potentially reveal the underlying biology or molecular phenotype of the tumor. For example, Buchelli et al. showed that the number of mitoses identified via a deep learning algorithm was predictive of the ODx risk categories [11].

Nuclear architecture is another image attribute that has been implicated in the prediction of overall cancer grade and cancer aggressiveness [12, 13]. Additionally, variations in nuclear shape could reflect genetic instability [14] and may impact the ability of cancer cells to travel through tissue and create metastases that lead to recurrence [15]. A number of recent studies have shown the association of QH features of nuclear architecture and morphology with disease progression in oropharyngeal cancers [16], cancer recurrence in lung cancers [17], biochemical recurrence in prostate cancers [18, 19] and overall breast cancer survival [20].

There is also evidence that the performance of QH analysis improves when done separately on different cell types [20]. In the context of distinguishing breast cancers with different degrees of risk, it is likely that these cancers are characterized by different phenotypical changes in different cell types. Breast cancers are predominantly carcinomas –cancers which are derived from epithelial cells [21]. In addition, there is evidence that stromal cells react to tumor growth over time, and stromal phenotype can reflect a given cancer’s genetic profile [22, 23]. For instance in [20], Beck et al. showed the importance of stromal morphology in predicting overall breast cancer survival. It is therefore useful to consider the behavior of epithelial and stromal cells as distinct groups when profiling breast cancer.

In this paper we evaluate the nuclear morphologic features to distinguish digitized images of H&E sections from early stage ER+ breast cancers into ODx risk categories using supervised machine learning classifiers. ODx risk categories are comprised of three groups to reflect distinctions based off 5 year survival: low, intermediate, and high risk [5, 24]. However, there is both a high degree of correlation between ODx risk categories and mBR grade [8], as well as overlap between the intermediate and low and intermediate and high risk categories, making accurate separation of intermediate cases from other risk categories difficult [25]. We have therefore selected four categories to distinguish using computer extracted nuclear morphology features: 1) Low ODx and Low mBR grade vs. High ODx and High mBR grade (Low-Low vs. High-High) to evaluate whether nuclear morphology features were able to predict risk category when both the difficult to classify intermediate cases and differences between mBR grade and ODx risk category are removed. 2) Low ODx vs. High ODx to evaluate the predictive ability of the nuclear morphology features when difficult to classify intermediate cases are removed. 3) Low ODx vs. Intermediate and High ODx to evaluate the ability of the nuclear morphology features to identify the low ODx cases specifically. 4) Low and Intermediate ODx vs. High ODx to evaluate the ability of the nuclear morphology features to identify high ODx cases specifically.

**Fig. 1:**
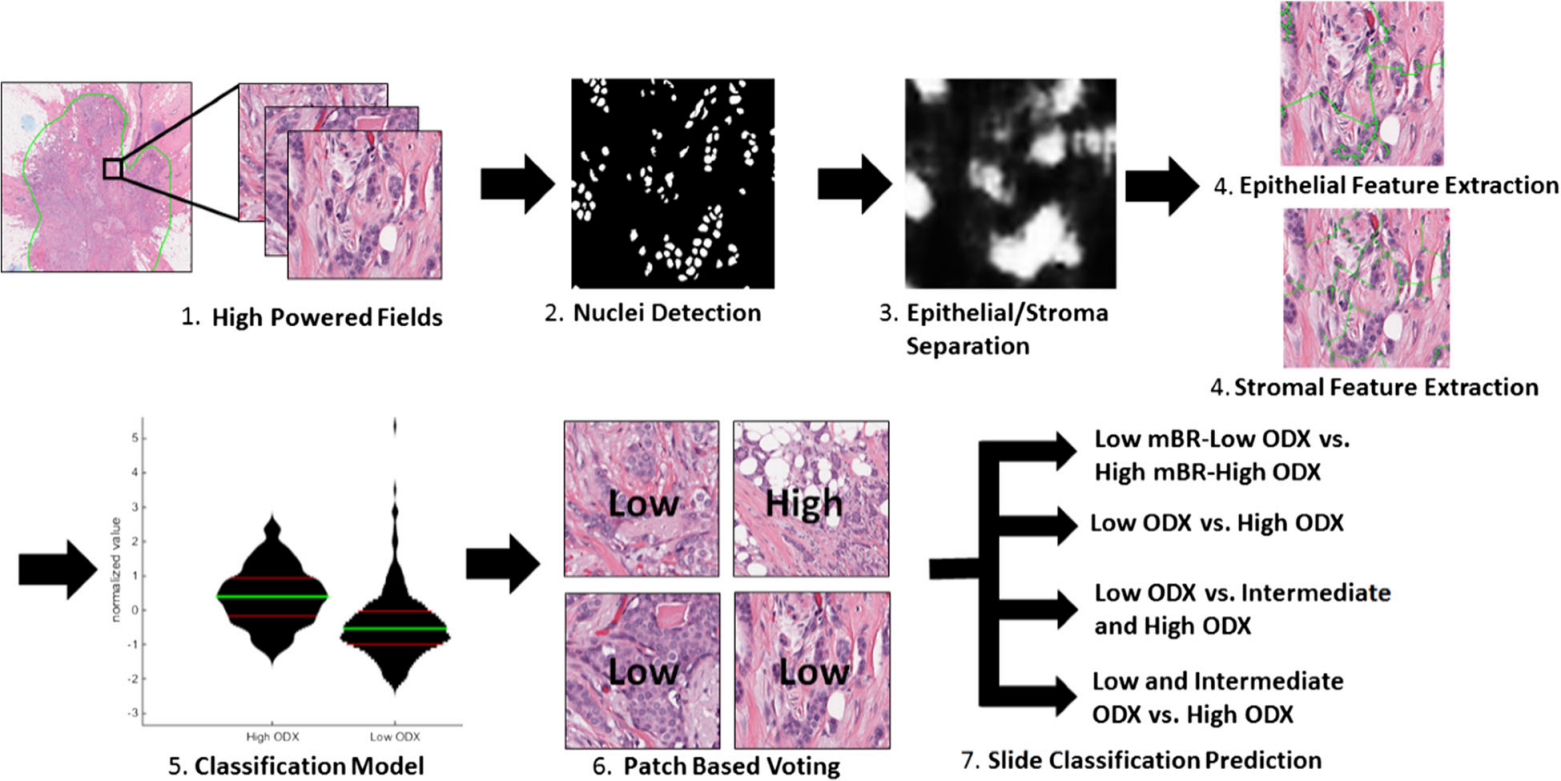
Illustration of the methodology used to classify whole slide images into ODx risk categories. 1) Image patches are extracted at 40× from regions within whole slides identified by pathologists as containing invasive cancer. 2) Nuclei detection is performed on these image patches and 3) combined with a Deep Learning epithelial/stromal separation model. 4) Nuclear architecture and shape features are extracted from the detected epithelial and stromal nuclei separately. These features are combined with (5) a trained classification model in order predict the ODx risk category for each patch. Classification results from the image patches for each patient are (6) combined in a patch-based-voting method to (7) yield the final risk prediction on a patient level

The approach presented in this paper comprises the following main steps (Fig. 1). First, H&E slides of surgical or biopsy specimens of breast tissue are scanned and digitized (Fig. 1.1). Second, nuclear segmentation is performed using deep learning models trained on manual breast nuclei annotations, followed by watershed separation to resolve overlapping nuclei (Fig. 1.2). Third, a deep learning model was used to separate epithelial from stromal regions, helping us identify which nuclei were stromal and which were epithelial (Fig. 1.3). Fourth, we extracted nuclear architectural and shape features from the epithelial and stromal regions separately (Fig. 1.4). Fifth, we perform feature selection on the resulting features using four different feature ranking schemes - Ranksum, PCA-VIP, MRMR MID, and MRMR MIQ. The predictive performance of these features was evaluated using four different supervised machine learning classifiers - random forest, support vector machine (SVM), linear discriminant analysis (LDA), and a neural network – via a 3-fold cross validation scheme (Fig. 1.5). The classifiers were evaluated by their ability to distinguish between the four different classification tasks presented above using the area (AUC) under the Receiver Operating Characteristic (ROC) curve, which plots the true positive rate against the false positive rate. Finally, classifiers are trained to create per-patch risk category predictions, identifying the optimal threshold of what percentage of positively classified patches should result in a positive prediction based on training data, and then applied and evaluated on testing folds to create a final prediction of the ODx risk category for each patient (Fig. 1.6, 1.7).

## Methods

### Dataset description

Our study comprised of 178 H&E stained whole tissue slides of ER+ Lymph node negative breast cancer patients (Table 1). These whole slide breast cancer samples dataset was selected to include 1) early stage ER+ breast cancers, 2) surgically resected tissue specimens, and 3) the availability of a corresponding Oncotype DX risk score. These slides were obtained from patients treated between 2004 and 2009 at the Cancer Institute of New Jersey and the University of Pennsylvania, and between 2008 and 2013 at Case Western Reserve University. Slides were locally digitized at their originating institutions using Aperio, Leica, and Philips scanners. The Modified Bloom-Richardson Grade for each of the pathologic specimens was determined by pathologists at each of the participating institutions. 9 cases in which the mBR score and ODx risk category were at opposite extremes (4 low mBR and High ODx, and 5 high mBR and low ODx) were excluded from this study.

**Table 1:** Dataset characteristics – demographic and cancer subtype distribution in each risk category for the cases from the 3 different institutions considered in this study

Parameters			
Oncotype DX Risk Category	Low (< 18)	Intermediate (> 18, ≤30)	High(> 30)
No. of Patients (*N* = 125)	66 (53%)	44 (35%)	15 (12%)
Age	20–77	25–70	45–70
Sex			
Female	66 (53%)	43 (34%)	15 (12%)
Male	0 (0%)	1 (1%)	0 (0%)
Patient Ethnicity			
White	33 (26%)	23 (18%)	5 (5%)
African American	3 (2%)	2 (2%)	2 (2%)
Asian	1 (1%)	2 (2%)	1 (1%)
Unknown	22 (18%)	17 (14%)	7(6%)
PR Status			
Positive	64 (51%)	39 (31%)	10 (8%)
Negative	2 (2%)	3 (2%)	5 (4%)
Unknown	0 (0%)	2 (2%)	0 (0%)
HER2 Status			
Positive	0 (0%)	1 (1%)	0 (0%)
Negative	66 (53%)	42 (34%)	15 (12%)
Unknown	0 (0%)	1 (1%)	0 (0%)
Histologic Tumor Grade			
Low (4, 5)	10 (8%)	14 (11%)	0 (0%)
Moderate (6, 7)	48 (38%)	24 (19%)	4 (3%)
High (8, 9)	8 (6%)	6 (5%)	11 (9%)
Tumor Type			
Ductal	53 (42%)	37 (30%)	14 (11%)
Ductal With Lobular Features	9 (7%)	3 (2%)	1 (1%)
Ductal with Mucinous Features	1 (1%)	2 (2%)	0 (0%)
Mixed	3 (2%)	2 (2%)	0 (0%)

### Nuclei segmentation

We employed the approach described in [26] by Janowczyk et al. for segmenting individual nuclei. Two Deep Learning (DL) models were employed. The first model identified the likelihood that a given pixel was part of a nucleus and the second model identified the likelihood that a pixel was part of the epithelium or stroma. Both models were trained using manual segmentations of the tissue primitives of interest (i.e. nucleus or stroma or epithelium). DL was executed using Caffe, a popular open-source DL framework [27]. The DL models were trained using 32 × 32 sized image patches on a Titan XGPU running CUDA 7.5, and a 9-layer convolutional neural network framework.

The nuclear segmentation model was trained on a dataset of 141 manually annotated ER+ breast cancer tissue images, each patch sized 2000 × 2000 pixels and at 40× magnification. The epithelium/stroma separation model was trained on a dataset of 236 ER+ breast cancer tissue image patches, each sized at 1000 × 1000 pixels and at 10× magnification. Lower magnification in the epithelial/stromal separation model allowed for more contextual information to be included in the image patches during model training, improving accuracy and speed. This patch-based approach allowed for multiple identically-sized image patches to be used, increasing the size of the training set. In addition, the patch size was selected to use the field of view identified as being optimal for extracting nuclear architecture features of the tumor [28].

### Feature extraction

A total of 216 nuclear features were extracted from epithelial and stromal nuclei separately, resulting in a total of 432 features per patch. These features consisted of architecture and shape features.

**Fig. 2:**
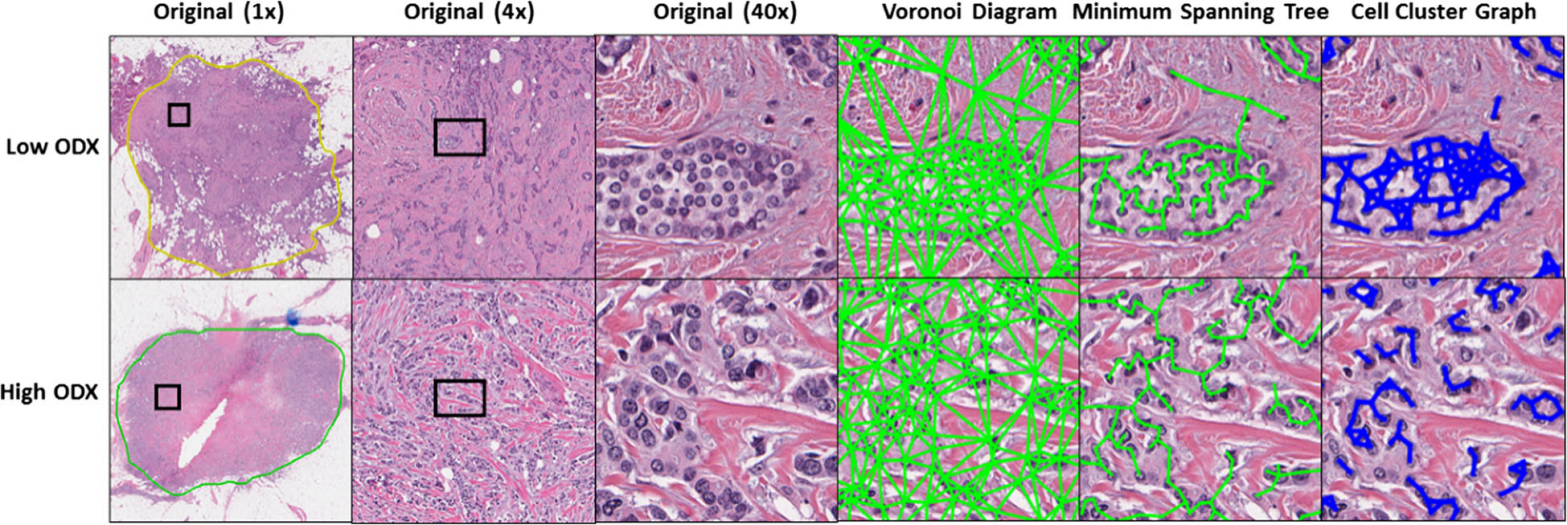
Nuclear graphs used to calculate features relating to spatial arrangement of nuclei. Left to right: Original images at 1×, 4×, and 40×, Voronoi Diagram, Minimum Spanning Tree, and Cell Cluster Graph, reflecting local nuclear architecture. Comparison between graph appearance for a low ODx example (top) and a high ODx example (bottom)

Architectural features were obtained by performing quantitative analysis of nuclear graphs, such as Delaunay Triangles, Voronoi Diagrams, Minimum Spanning Trees (MST), and Cell Cluster Graphs (CCG) [29] (Fig. 2). These nuclear graphs were constructed using the individual nuclei as the vertices of the graph. The choice of vertex connectivity determines the type of nuclear graph (i.e. Delaunay, Voronoi, MST, CCG) constructed. Features extracted from the graphs included changes in the lengths of edges and distance between nearest vertices. Cellular disorder can be measured using features derived from Cell Orientation Graphs [19]. Shape features included Invariant Moment, Fourier Descriptor, and Length/Width ratios. A comprehensive enumeration of all the image features extracted is presented in the Additional file 1.

### Feature ranking

Feature ranking was used to identify the most relevant image features for predicting the corresponding ODx risk category. Features were ranked in order of highest relevance to the classification problem. The most relevant features identified were subsequently used in conjunction with machine learning classifiers. A number of popular feature ranking methods were evaluated including Wilcoxon Ranksum [30], PCA-VIP [31], and Maximum-Relevance Minimum-Redundancy (MRMR) [32] with two variants – Mutual Information Difference, and Mutual Information Quotient (MRMR-MID and MRMR-MIQ) [33]. Each of these feature ranking methods takes a slightly different approach to identifying the most relevant features, and simultaneously suppressing features that are highly correlated with each other. The Ranksum method identifies feature relevance to classification without explicitly considering the correlation between highly-ranked features [30]. PCA-VIP uses a combination criteria of both how each of the principle component vectors relate to the outcome to be predicted, and which features most highly contribute to those principle component vectors (effectively measuring to what extent a given feature provides unique information in a dataset) [31]. MRMR-MID and MRMR-MIQ both use maximal relevance criteria which use the mean mutual information values between features and the relevant output class, while minimizing the redundancy (mutual information between any feature and the other features in the dataset) [32].

### Classifier construction

**Fig. 3:**
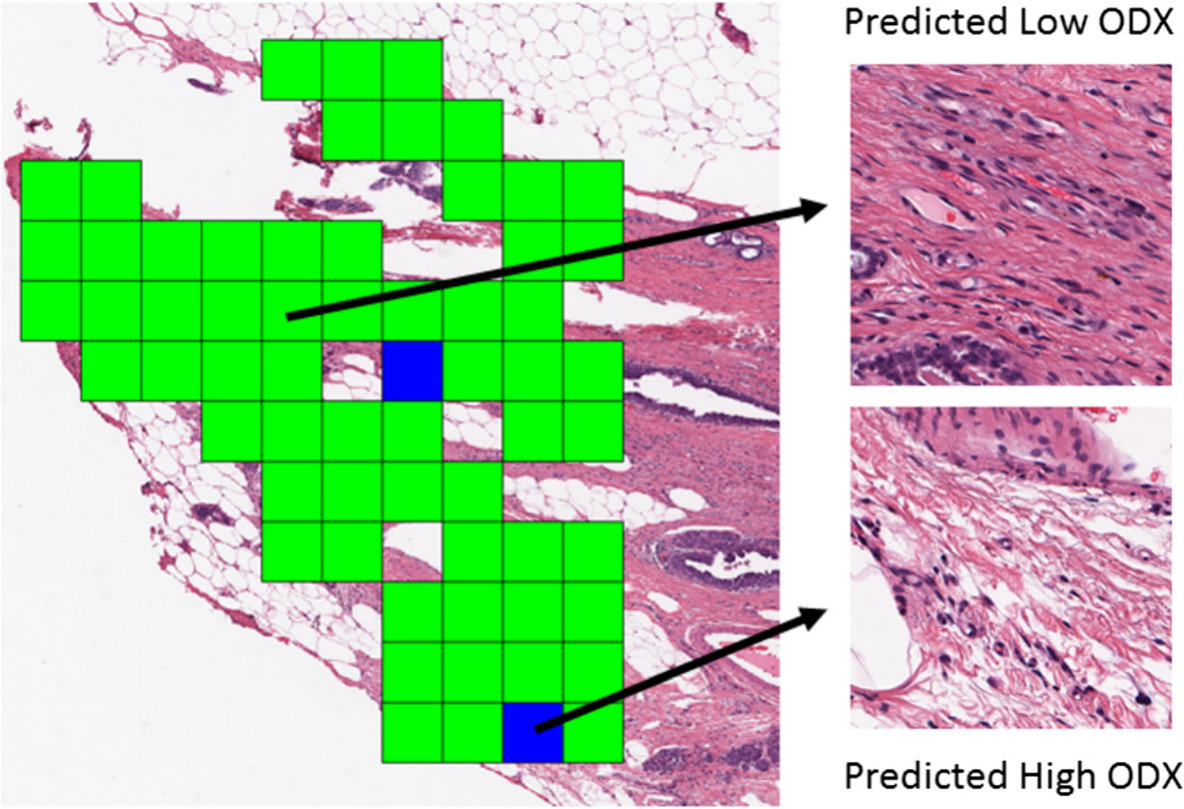
Example of the Low-Low vs. High-High random forest classifier using ranksum feature selection applied to patches from whole slide image. Machine classification uses the top ranked epithelial and stromal features. Green squares indicate patches that are predicted to be Low ODx while Blue squares are predicted to be High ODx

A total of four different classifiers was tested in conjunction with each of the four different feature selection methods. The classifiers employed included a bagged C4.5 Random Forest [34], a ten-node four-layer Neural Network [35], a 3 kernel Support Vector Machine [36], and a pseudolinear discriminant Linear Discriminant Analysis [37]. Machine learning classifiers were trained using 100 iterations of randomly initialized 3-fold cross-validation. 3-fold cross-validation was employed to divide the entire dataset of image patches into three equal groups by patient ID, thus ensuring that patches from each patient were not simultaneously present in the training and hold-out groups. Two of these groups were used for model training, while the third group was used to test the trained model. Machine learning classifiers were trained on a per-patch basis. This allowed for a simple patch-based voting method, in which the classification of the patient as being in the low or high-risk category was based on if the number of class labels predicted for a given class surpassed a patch percentage threshold. The optimal threshold was determined from the training data in each iteration. This method can also be used to classify individual patches spatially in an H&E slide, providing a spatially distributed assessment of cancer aggression across a given sample (Fig. 3).

### Experiments

#### The four experiments were as follows

1. Low ODx and Low mBR grade vs. High ODx and High mBR grade (Low-Low vs. High-High). This experiment was used to look at the cases reflecting the extremes in terms of tumor morphology and ODx risk. While grade and ODx risk scores are correlated for the most part [8], in this experiment we chose to ignore conflicting cases (i.e. cases with a low mBR grade but a high ODx score and vice-versa).
2. Low ODx vs. High ODx. This experiment looks at cases of high distinction in terms of ODx risk category, but does not exclude cases with conflicting grade categories.
3. Low ODx vs. Intermediate and High ODx. This is the hypothesis that is closest to the question a clinician is interested in answering: identifying cases that are low ODx risk score from all others so that low ODx risk patients can avoid aggressive chemotherapies.
4. Low and Intermediate ODx vs. High ODx. This experiment considers the possibility that high ODx risk patients are histologically distinct from both other ODx risk categories.

We also quantitatively assessed the performance of each of four different feature ranking methods over stromal and epithelial features in conjunction with four different machine learning classification schemes to determine which combination of classification and feature ranking approaches resulted in the highest per-patient patch voting accuracy for each of the four experiments. Per-patient patch voting simply means that the classifier was applied to each patch extracted from a patient, thus generating an ODx risk category prediction for each patch. A simple majority of the per-patch risk category predictions for each patient is then used to determine the predicted patient ODx risk category. The per-patient patch voting accuracy is defined as the percentage of patients whose ODx risk category was correctly predicted using this method.

### Feature evaluation via supervised classification

**Fig. 4:**
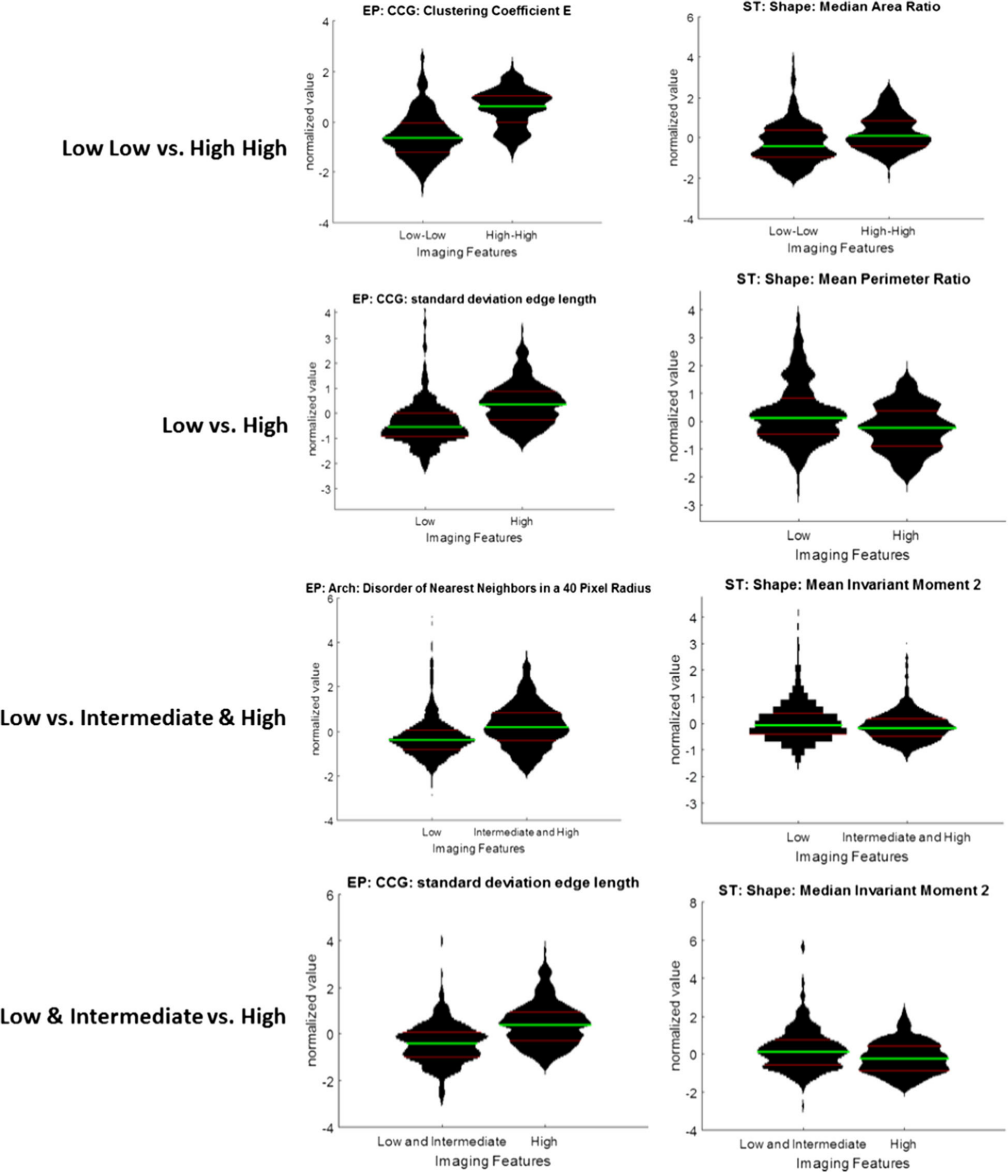
Feature Distributions for the top ranked epithelial (left) and stromal (right) features using PCA-VIP feature ranking for each experiment. Green lines indicate the mean of each population, and red lines indicate the 25th and 75th percentiles of the distribution. Width of the plot indicates the relative number of data points at each normalized feature value along the y-axis

For each of the 4 classification experiments described above, we identified 1) the most highly ranked and predictive epithelial and stromal nuclear morphologic features which were evaluated via violin plots (Figs. 4), and 2) classification accuracy for the machine learning classifiers in conjunction with the top ranked features in the form of AUC.

Violin plots illustrate the distribution of normalized feature values for the top performing features between the two risk categories. Thus, high degrees of separation between the two distributions indicate a high level of discrimination from that feature. AUC curves indicate the true positive rate as a function of the false positive rate at varying confidence thresholds. The higher the area under the curve (indicated by the curve extending into the upper left quadrant), the more frequently the classifier is able to correctly identify the class, and the less frequently it is to falsely classify a case as positive. For comparison, a diagonal line extending from the bottom left to the upper right corner would indicate an AUC of 0.5, which is considered to be the equivalent of guessing.

In order to demonstrate the significance of epithelial/stromal separation, we ran two sets of features using the optimized machine learning classifier and feature ranking algorithm. The two feature sets were: 1) nuclei features extracted from all nuclei, 2) nuclei features extracted from epithelial and stromal nuclei separately. The utility of separating epithelial and stromal nuclei prior to feature extraction was measured by comparing the AUCs between models trained from features with no epithelial/stromal separation, and epithelial stromal separation prior to feature extraction.

### Evaluation of models on external validation set

In order to fully assess the effectiveness of the models generated, the models with the highest performance were used on an external validation set. Models were trained over the entire primary cohort before being applied without any retraining to the external validation set.

## Results

### The results for the four primary experiments are as follows

**Fig. 5:**
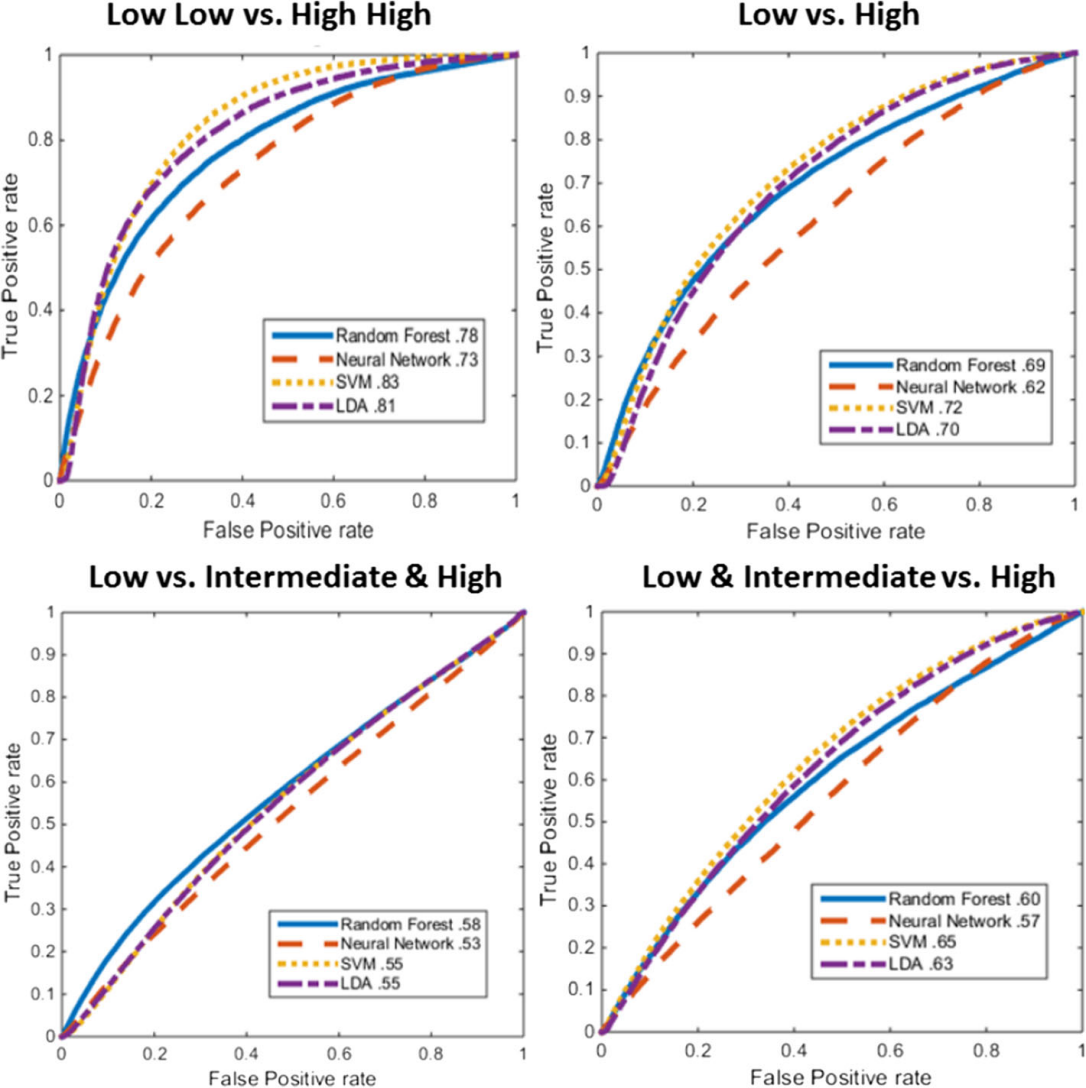
ROC curves for each of the four experiments conducted (panels) and classification methods (lines) using PCA-VIP feature selection. Top left: Low ODx and Low mBR grade vs. High ODx and High mBR grade (Low-Low vs. High-High). Top Right: Low ODx vs. High ODx. Bottom Left: Low ODx vs. Intermediate and High ODx. Bottom Right: Low and Intermediate ODx vs. High ODx. Each panel displays the ROC curve using either (solid) random forest, (dashed) neural network, (dotted) SVM, or (intermediate dash) LDA classification. Feature set includes epithelial and stromal features. AUC values for each curve are displayed in the legend

1. Low ODx and Low mBR grade vs. High ODx and High mBR grade (Low-Low vs. High-High) (Fig. 5, top left). In this experiment, the top ranked epithelial features were cell cluster graphs, and the top ranked stromal features were shape features related to nuclear perimeter, area ratios, and invariant moment (Table 3). The SVM classifier using the PCA-VIP feature ranking scheme yielded the highest classification accuracy with an AUC of 0.83, and a patch voting accuracy of 86% (Table 2). AUC results using the same classifier and feature ranking methodology improved from 0.71 to 0.83 with the inclusion of stromal features (Table 4).
2. Low ODx vs. High ODx (Fig. 5, top right) (Fig. 5, top right): The top ranked epithelial features were the cell cluster graph and disorder of nearest neighbors features, while the highest ranked stromal features were similar to those identified for the low-low vs. high-high discrimination problem, namely perimeter ratio, area ratio, and invariant moment (Table 3). The SVM classifier using the PCA-VIP feature ranking scheme yielded a classification AUC of 0.72, and a patch voting accuracy of 76% (Table 2). AUC results using the same classifier and feature ranking methodology improved from 0.61 to 0.72 with the separation of epithelial and stromal nuclei (Table 4).
3. Low ODx vs. Intermediate and High ODx (Fig. 5, bottom left): The top ranked epithelial features were primarily disorder and number of nearest neighbors features, while the highest ranked stromal features were primarily metrics regarding the invariant moment (Table 3). The random forest classifier using the PCA-VIP feature ranking scheme yielded a classification AUC of 0.58, and a patch voting accuracy of 64% (Table 2). AUC results using the same classifier and feature ranking methodology improved from 0.55 to 0.58 with the separation of epithelial and stromal nuclei (Table 4).
4. Low and Intermediate ODx vs. High ODx (Fig. 5, bottom right):: The top ranked epithelial features were metrics concerning the mean and variation in edge length associated with cell cluster graphs, while the highest ranked stromal features were the invariant moment and standard deviation of the Fourier descriptor (Table 3). The SVM classifier and PCA-VIP feature ranking scheme yielded an AUC of 0.65, and a patch voting accuracy of 74% (Table 2). AUC results using the same classifier and feature ranking methodology improved from 0.55 to 0.65 with the separation of epithelial and stromal nuclei (Table 4).

**Table 2:** Classification accuracy metrics for each of the four experiments. From left to right: Low ODx Low mBR vs. High ODx Low mBR, Low ODx vs. High ODx, Low ODx vs. Intermediate and High ODx, and Low and Intermediate ODx vs. High ODx. Data for each experiment includes the AUC, best patch Voting Accuracy results, and the optimal feature ranking and classifier used to achieve the optimized patch voting accuracy results. All experiments conducted with 3-fold cross-validation

Experiment	LL vs. HH	L vs. H	L vs. Int. and H	L and Int. vs. H
Number of Patients	37	75	125	111
AUC	0.81	0.69	0.58	0.6
AUC STDev	0.08	0.05	0.03	0.06
Patch Voting Accuracy	82%	80%	60%	86%
Best Feat. Ranking for Patch voting	MRMR-MID	PCA-VIP	Ranksum	MRMR-MID
Best Classifier for Patch voting	LDA	Random Forest	Random Forest	Random Forest

**Table 3:** Top three Epithelial and Stromal features for each of the four experiments: Low ODx and Low mBR grade vs. High ODx and High mBR grade (Low-Low vs. High-High), Low ODx vs. High ODx, Low ODx vs. Intermediate and High ODx, and Low and Intermediate ODx vs. High ODx

Experiments	Epithelial Features (EP)	
Low Low vs. High High	1	EP: CCG: Clustering Coefficient E
	2	EP: CCG: standard deviation edge length
	3	EP: CCG: Clustering Coefficient D
Low vs. High	1	EP: CCG: standard deviation edge length
	2	EP: CCG: mean edge length
	3	EP: Arch: Disorder of Nearest Neighbors in a 40 Pixel Radius
Low vs. Intermediate and High	1	EP: Arch: Disorder of Nearest Neighbors in a 40 Pixel Radius
	2	EP: Arch: Disorder of Nearest Neighbors in a 50 Pixel Radius
	3	EP: Arch: Avg. Nearest Neighbors in a 40 Pixel Radius
Low and Intermediate vs. High	1	EP: CCG: standard deviation edge length
	2	EP: CCG: mean edge length
	3	EP: Arch: Disorder of Nearest Neighbors in a 40 Pixel Radius
	Stromal Features (ST)	
Low Low vs. High High	1	ST: Shape: Median Area Ratio
	2	ST: Shape: Median Invariant Moment 2
	3	ST: Shape: Mean Perimeter Ratio
Low vs. High	1	ST: Shape: Mean Perimeter Ratio
	2	ST: Shape: Mean Area Ratio
	3	ST: Shape: Mean Invariant Moment 2
Low vs. Intermediate and High	1	ST: Shape: Mean Invariant Moment 2
	2	ST: Shape: Median Invariant Moment 2
	3	ST: Shape: Standard Deviation Invariant Moment 2
Low and Intermediate vs. High	1	ST: Shape: Median Invariant Moment 2
	2	ST: Shape: Mean Invariant Moment 2
	3	ST: Shape: Standard Deviation Fourier Descriptor 2

**Table 4:** Improvements in classification accuracy based on features extracted from all nuclei together (No Ep/St. Sep.) vs. features extracted from epithelial nuclei and stromal nuclei separately (Ep/St Sep.), ranked via the PCA-VIP feature selection scheme, and used to train an SVM classifier. All AUC scores were generated using 3-fold cross validation

Experiment	No Ep/St Separation	Ep/St Separation	AUC Improvement
High-High vs. Low-Low	0.71	0.83	0.12
High vs. Low	0.61	0.72	0.11
Low vs. Intermediate and High	0.55	0.58	0.03
Low and Intermediate vs. High	0.55	0.65	0.1
Average	0.61	0.7	0.09

**Fig. 6:**
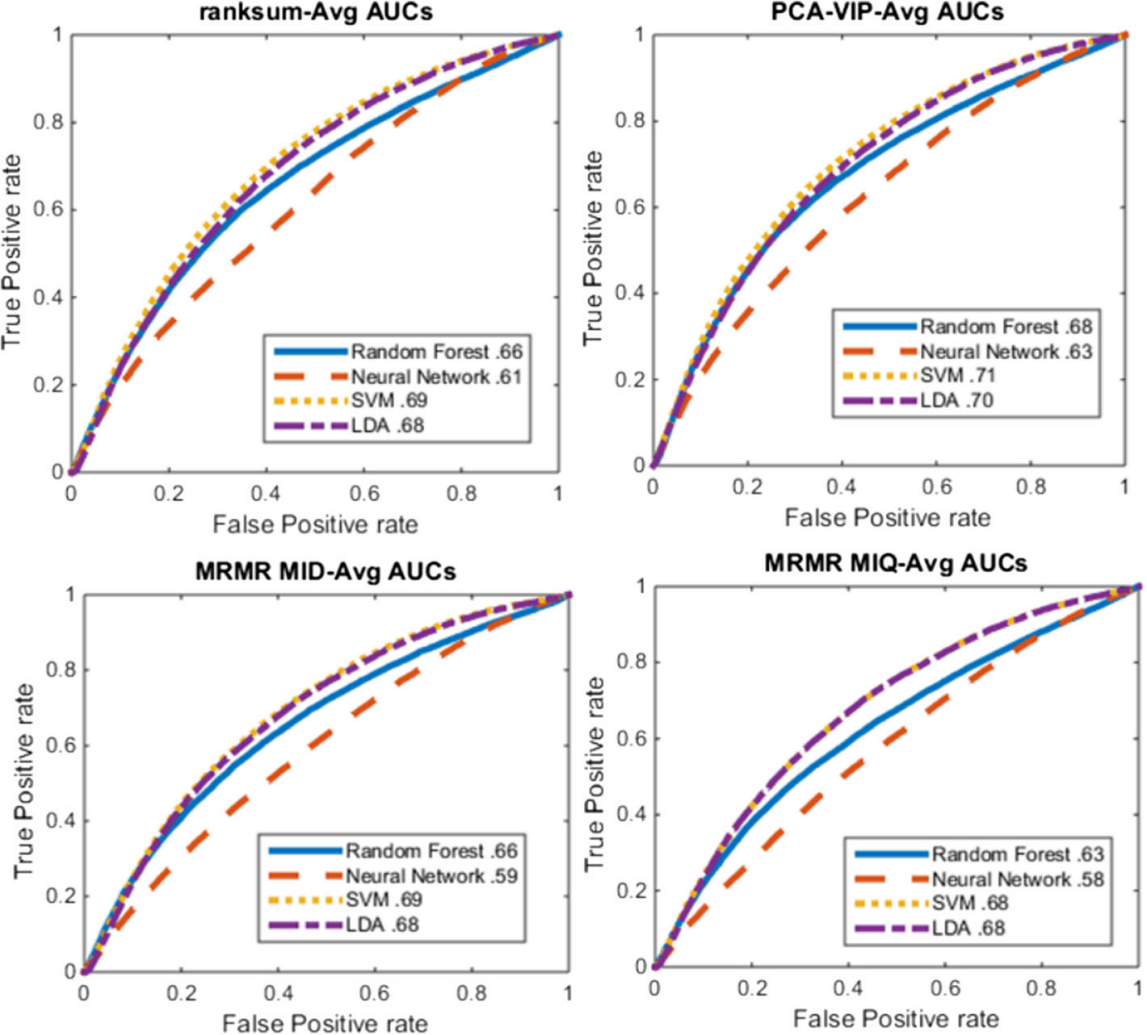
Determining the optimal feature ranking method - ROC curves for different combinations of feature ranking methods (panels) and classification methods (lines) for separating low from high ODx patches. Top left: Ranksum (Wilcoxon rank sum). Top right: PCA-VIP. Bottom left: MRMR-MID. Bottom right: MRMR-MIQ. Each panel displays the ROC curve using either (solid) random forest, (dashed) neural network, (dotted) SVM, or (intermediate dash) LDA classification. Feature set includes stromal and epithelial features. AUC values for each curve are displayed in the legend

Of the epithelial features considered, the most discriminating features identified across all 4 classification problems were those pertaining to epithelial architecture of nuclei (Table 3). Of the stromal features, the most significant tended to be those related to measuring changes in the shape of the stromal nuclei. In each experiment, the epithelial features were identified to be more significant in separating the different risk categories compared to the stromal nuclei features (Fig. 6). The classification AUC for the machine learning classifier was highest for the problems involving the extreme risk or grade categories (i.e. Low-Low vs High-High and Low ODx vs High ODx). Unsurprisingly, the AUC values were lower when the intermediate risk category was also included (i.e. Low ODx vs. Intermediate and High ODx and Low and Intermediate ODx vs. High ODx).

In addition, while each of the feature ranking methods had very comparable performance, the PCA-VIP feature ranking scheme yielded slightly better performance, with a peak AUC of 0.71 using a Support Vector Machine (Fig. 6).

Comparisons between the classification efficacy with and without the use of epithelial/stromal separation across the four experiments yielded an average improvement of 0.09 (Table 4).

### Validation results

We tested the results of the model on an external validation set. The model was trained using Ranksum feature ranking and a Random forest classifier using 100 iterations of 3-fold cross-validation to determine the top-performing features. These features were then trained over the entire training set before being evaluated on the validation set. The validation set was obtained from the University of Pennsylvania and contained 53 cases comprised of Low and High ODx risk cases of primarily Low and High mBR grade (Table 5). As described previously, the accuracy of each model was determined using per-patient patch voting, where pathologist selected ROIs were divided into sub-ROI patches, and each patch was then classified as belonging to either low or high risk using each of the four models. The classification of the patient into high or low risk was determined by the percentage of sample patches predicted to belong to either category. Because it is possible that the optimal percentage threshold for distinguishing between high and low risk may not be a simple majority, the ideal percentage of patches that were need to be identified as low for the patient to be categorized as low ODx risk was determined from the training set. Per-patient accuracies ranged between 76 and 85% across all hypotheses evaluated. Improvements in classification accuracy of low vs. high over low-low vs high-high may be explained by the fact that the validation set was composed exclusively of low and high ODx samples. In addition, the larger number of samples which were low ODx as compared to high ODx samples may explain why the model trained to distinguish between low and intermediate vs high had slightly improved performance over the model trained to distinguish between low vs. intermediate and high. It may also reflect the fact that the low and intermediate risk patients are more alike from a histomorphometric perspective compared to the intermediate and high risk patients. The accuracies were highest using models trained to distinguish between Low vs. High and Low vs (Intermediate and High ODx) cases (Table 6).

**Table 5:** Validation dataset characteristics – ODx and grade distribution

Validation Set (*N* = 53)			
mBR Tumor Grade\ODx Category	Low (< 18)	Intermediate (> 18, leq30)	High (> 30)
Low (4, 5)	40 (75%)	0 (0%)	0 (0%)
Moderate (6, 7)	0 (0%)	0 (0%)	1(2%)
High (8, 9)	0(0%)	0 (0%)	12 (23%)

**Table 6:** Validation dataset – Classification accuracy using Ranksum feature ranking and a SVM classifier for each of four classification separations

Ranksum - SVM & Classification Accuracy	
Low-Low vs. High-High	76%
Low vs. High	79%
Low and Intermediate vs. High	85%
Low vs. Intermediate and High	84%

## Discussion

In this work, we evaluated the effectiveness of computer-extracted measurements of size, shape, and architectural features of epithelial and stromal nuclei in separating early stage ER+ breast cancer histology samples into different Oncotype DX determined risk categories. Nuclear feature extraction was accomplished by 1) obtaining nuclear segmentations with a deep learning algorithm, 2) using deep learning epithelial/stromal separation of nuclei, and 3) extracting nuclei shape and architectural features from those segmentations. Those features were then given to a series of machine based classifiers and feature ranking methods using 3-fold cross-validation to test the effectiveness of each machine based classifier. These features were then employed in the context of discriminating the following 4 different grade-ODx risk categories: 1) Low ODx and Low mBR grade vs. High ODx and High mBR grade (Low-Low vs. High-High). 2) Low ODx vs. High ODx. 3) Low ODx vs. Intermediate and High ODx. 4) Low and Intermediate ODx vs. High ODx.

We found that the best classifier accuracy (AUC = 0.83) was obtained for the Low-Low vs. High-High classification problem. Since the ODx risk category is strongly correlated with tumor grade [9], by choosing to leave out conflicting cases (i.e. where the grade and ODx risk categories are not aligned), the Low-Low vs High-High categories represent the extreme risk cases. The next highest accuracy was obtained for the Low ODx vs. High ODx categories, where all intermediate risk cases were left out. The best classifier AUC obtained in this experiment (AUC = 0.72) was lower compared to the AUC obtained for the Low-Low vs High-High problem, possibly due to presence of 64 cases (55 Intermediate mBR and Low ODx, and 9 Intermediate mBR High ODx) where the grade and ODx risk categories did not align. This most likely adversely affected the training and the evaluation of the machine learning classifiers. When evaluating the classifiers in distinguishing the Low vs. Intermediate and High and the Low and Intermediate vs. High ODx risk categories, the Low and Intermediate vs. High ODx distinction had slightly improved performance as compared to distinguishing Low vs. Intermediate and High ODx risk categories. This may be due to the fact that the intermediate cases identified by ODx were primarily low risk cases [38].

Classifier models trained on Low vs. High and the Low with Intermediate vs. High ODx cases yielded the highest classification accuracy on the validation set. These results appear to suggest that histomorphometrically the low ODx and intermediate ODx appeared more similar compared to the high ODx cases. Clearly this will need to be validated in additional, larger independent validation studies, but if confirmed might suggest that a number of the patients currently classified as intermediate risk by Oncotype DX might actually be low risk and should be classified as such.

Tumor grade is determined by tubule formation, nuclear pleomorphism, and mitotic count [39]. These same features are found to strongly correlate breast cancer outcome [40]. The state of tubule formation is reflected in features such as the ratio of tubule nuclei to total nuclei [41]. The architecture of tubule formation is also reflected in features used in the presented work, such as Cell Cluster Graphs [29], Cell Orientation Entropy [19], and Disorder of Nearest Neighbors [19]. Nuclear pleomorphism may be reflected in features such as the Mean Invariant Moment [42], and Area Ratio [43]. Thus, the features used in this work are implicitly reflective of the histomorphometric measurements used by pathologists to assess grade and breast cancer outcome. However, the method presented can also identify complex and sub-visual (i.e. information which is present, but not easily discernable by a human, such as higher-order nuclei architectural characteristics, or difficult to recognize chromatin patterns [44, 45]) relationships between quantitative features and ODx categories that are difficult for pathologists to visually identify. The Oncotype gene expression test aims to capture changes in genetic expression in genes that have been tied with specific cancer-related traits [46]. For example, Ki-67, STK15, Survivin, Cyclin B1, and MYBL2 have all been associated with breast cancer proliferation; Stromelysin 3 and Cathepsin L2 have been associated with invasion; and ER, PR, Bcl2, and SCUBE2 have been associated with responsiveness to Estrogen [47]. Variations in these genes could potentially lead to changes in visual presentation of the cancer, and thus affect the features previously described. For example, increases in Ki-67 activity resulting in increased unregulated cell proliferation may increase the density of cell nuclei, resulting in an increase in the Disorder of Nearest Neighbors, or decreased distance between nuclei in Cell Cluster Graphs. Tumor invasion resulting from activation of Stromelysin 3 could result in either a loss of tissue differentiation, or the presence of large epithelial nuclei invading into the surrounding stroma [48]. These types of phenotypic changes might be captured by architectural features, or size and shape variation amongst stromal nuclei features. For example, variation in stromal nuclei shape could also be related to the connection between spindle-cell and round stromal nuclei contact and breast cancer patient survival discovered by Beck et al. [20].

Previous groups have been able to duplicate ODx results using equations drawing from genetic expression and pathologist grading information, such as the Magee Equation [9]. Using these methods, low grade and low ER and PR (≤150) can be correctly categorized as being low ODx 89% of the time; and when ignoring intermediate ODx cases, low and high ODx samples can be correctly identified with concordance rates between 96.9 and 100% [25, 49]. However, these methods have between 54.3 and 59.4% concordance when considering intermediate cases as well as low and high, and require pathologist-generated data [25]. When considering the intermediate risk categories, our classification AUC ranged from 0.58 and 0.6 which appears to be in alignment with the findings in [25].

Several different groups have previously explored the use of QH for predicting ODx risk categories. For example, Basavanhally et al. was able to separate high from low grade breast cancer patients, with top performing architectural features such as Delaunay Triangle metrics, nuclei density, and Voronoi Diagram architectural information [12]. Romo-Bucheli et al. was able to separate high-high from low-low cases with an AUC of 0.76 using a single feature: the ratio of tubule nuclei to non-tubule nuclei [41]. This approach used Deep Learning to identify biologically relevant structures (separating tubule nuclei from non-tubule nuclei), while the presented approach used a much larger number of nuclei-specific features for classification purposes.

While related to these previous approaches [12], our focus was on quantitatively evaluating the role of computer extracted features of nuclear morphology in the stroma and epithelium with the Oncotype Dx risk categories. Additionally, unlike previous related studies [13] our study looked at the most discriminating features to distinguish not just the extreme risk categories (low vs. high) but also looked at the ability of computer extracted nuclear morphologic features to distinguish the intermediate risk categories from the low and high risk categories.

We do however acknowledge the several limitations of this work. Firstly, the validation set used only included high and low ODx cases, without any intermediate cases. Secondly, the focus of this work was on finding features that were associated with ODx risk categories and not patient outcome. Oncotype DX is a companion diagnostic test, and while the risk categories have been validated against outcome, it is not perfectly correlated [50]. Unfortunately, long-term disease recurrence or patient outcome information was not available for the cases considered in this study. We also did not conduct a detailed study of the influence of staining and scanning variations on the features identified as predictive and the influence of these parameters on the subsequent classification results. Finally, we focused solely on the role of nuclear morphology in this work, there are clearly other features that are known to have a prognostic role in early stage ER+ breast cancers, features relating to number and distribution of tumor infiltrating lymphocytes, mitoses [11], and tubules [41]. These features have shown to be independently useful in determining ODx risk categories in ER+ breast cancer, and would likely improve the classification results when combined with the nuclear histomorphometric features presented in this work. Another potential future avenue is the integration of histomorphometric approaches such as this with genomic based tests to determine if the integration of morphologic and molecular measurements enables more accurate risk assessment, especially for the patients currently identified as intermediate risk. We hope to address these limitations in future work.

## Conclusions

In this work we evaluated the role of computer extracted features relating to spatial architecture and shape within the epithelium and stroma and showed that these features could distinguish early stage ER+ breast cancers into different ODx risk categories. Our results suggest that with additional validation, these features could be used to create an inexpensive, rapid, and nondestructive predictor of low and high ODx risk categories for early stage ER+ breast cancer based off digitized images of H&E slides alone.

## Additional file

Additional file 1: **Table S7.** Features tested for significance, and considered for use in final analysis. Comprehensive list of features investigated for classification utility. Each feature was used to analyze epithelial and stromal nuclei separately. (XLSX 15 kb)

## Abbreviations

CCG: Cell Cluster Graph
ER +: Estrogen Receptor Positive
H&E: Hematoxylin and eosin
LDA: Linear Discriminant Analysis
mBR: Modified Bloom-Richardson
MRMR MID: Maximum Relevance, Minimum Redundancy, Mutual Information Difference
MRMR MIQ: Maximum Relevance, Minimum Redundancy, Mutual Information Quotient
MST: Minimum Spanning Trees
ODx: Oncotype Dx
PCA-VIP: Primary Component Analysis – Variable Importance
QH: Quantitative Histomorphometry
ROC: Region Under the Curve
SVM: Support Vector Machine
